# Indents

**Published:** 1905-04-15

**Authors:** 


### INDENTS.

**☞ Brief Articles of Technical or Scientific Value will receive notice and comment. Contributions invited.**

**Rusty Instruments.** Place the instruments in a saturated solution of zinc chlorid over night, and the rust will be destroyed by reduction. Rinse in hot water and wash in hot soap and soda solution. Polish with alcohol and chalk.—*Pac. Med. Record.*

**Low-fusing Alloy.** Take five parts of bismuth, and one and one-half parts of cadmium, and two parts of tin and melt together. This alloy will fuse so low that it can be poured directly into a plaster impression. It produces an accurate die for shot swaying or any similar process.—W. A. Busch, Chicago.

**Retaining Dentures.** Dr. Wm. M. Gabriel, in the *Dental Record,* says if possible, no mouth should ever be made edentulous. If one or two roots only can be kept they will serve an excellent purpose in steadying a plate. A good plan is to fit such roots with a gold crown and tube into which a pin may be projected from a plate.

**Eucain-Adrenalin Local Anesthesia.** Freund relates his experience with local anesthesia with a mixture of two parts of 1 per 1,000 adrenalin to 8 parts of a 1 percent solution of beta eucain, injected under the skin or mucosa or both. He regards it as indispensable for prolapse operations and for plastic operations on the perineum. No by-effects were ever observed in his extensive experience except that a few extra sensitive patients complained of transient smarting.—*Jour. A. M. A.*

**Porcelain vs. Gold.** I am perhaps as enthusiastic and favor the use of porcelain quite as much as any one present, but I still use gold, and still place my dependence on gold in many cases; with us I think it will be a long time before gold will be displaced as an agent for saving teeth in those cases where we can use it without making a blemish in the mouth.—W. D. Tracy, in *Cosmos*.

**Obtunding Dentin.** Dissolve ten grains of nervocidine in two drams of the fluid of zinc oxysulphate cement, and mix with the powder as much as may be needed to cover all the exposed dentin, but not enough to fill the cavity, and seal to place with gutta-percha temporary stopping. In from five to six hours the cavity may be excavated painlessly.—F. Auguilar, in *Review*.

**Cleaning a Hypodermic Needle.** If stopped, remove the needle from the syringe and pass it through a piece of unvulcanized rubber. Fill the syringe with water and insert the needle point first until the vulcanite packing is forced against the hub. By holding the needle firmly into the syringe and making pressure the debris will be forced out of the needle.—C. W. Kraemer, in *Review*.

**Ethyl Chlorid Anesthesia.** Hilliard describes the method of using ethyl chlorid, its dose, after-effects, clinical signs, and the preparation and posture of the patient. He considers this preparation an admirable anesthetic, either alone or with air, with nitrous oxid, with chloroform, with ether, with A. C. E. mixture, and as a preliminary to each of the last three for a large variety of surgical operations. It is contra-indicated in diseases of the larynx, inflammatory lesions and tumors in, or adjacent to, the respiratory passages; goiter; all conditions giving rise to urgent dyspnea, and in long operations.—London *Lancet*.

**Root Filling Material.** Make a saturated solution of thymol in eucalyptus, and dissolve in one ounce of it one-half ounce of base-plate gutta-percha. The gutta-percha should first be dissolved in chloroform and then mixed with thymol and eucalyptus. Let the chloroform evaporate and the paste is ready for use. It can be worked into the canals with a warm brooch, and forced into all the canal with a hard gutta-percha cone.—B. L. Cochran, in *Review*.

**Dentist a Mechanic.** January 5th, a judge at Nashville, Tenn., had to decide the status of a dentist. The case grew out of a dentist having his instrument levied upon. He replevined them, and the man who made the original levy insisted that they did not come under the exemption—"necessary in the pursuit of trade"—as is the case with a mechanic's tools, but the judge decided that a dentist was a mechanic and that his tools were consequently exempt.—*Digest*.

**Dentist not Responsible for Loss.** Last month a judge in the First District Court at Newark, N. J., handed down a decision which is of interest. A woman took a set of false teeth to a dentist to be repaired. While in his hands they disappeared, and according to the court's decision the inference was that they had been taken by an employe. The woman sued to recover the value, but the court held that the dentist had used all due care and was not responsible for their loss.—*Digest*.

**Greater Pneumonia Mortality in Flats.** The insanitary possibilities of the slum tenement house are sufficiently well known. Those of the average medium-priced flat we have already dilated on to some extent. Now it is reported that the pneumonia mortality is especially high in the high-priced apartment buildings—the tenements of the rich—in New York City. Every phase of excessive urban concentration seems to have its special unhygienic accompaniments.

**Excision of the Semilunar Ganglion in Facial Neuralgia.** Dr. A. E. Halstead, Chicago, reported seven cases. The pain may return after the removal of the ganglion. In his cases each ganglion was carefully examined by a competent microscopist. In each ganglion elements were found, and the gross anatomic characteristics of the organ were preserved. Dr. John B. Murphy stated that in his twelve cases there were four deaths. This large percentage caused him to abandon the operation. Since his last report he has had only one recurrence of neuralgia after the injection of osmic acid.

**Bandless Porcelain Crown.** The article in the December *Digest* on "The Bandless Porcelain Crown," by Dr. R. A. Pritchett, interested me, as I agree with him. My method of setting the crown is an improvement on cement alone. When the crown is fitted to the root and properly shaped, take a small portion of temporary stopping, heat and mold around the base of the post, press the crown to place, remove and dress off surplus, reheat and return to position, allow to cool, remove again, trim off any surplus, also any extending into hole in root, and cement to place. The temporary stopping is insoluble and seals the cement, preventing the fluids of the mouth from disintegrating it. —John G. Harper, in *Digest.*

**Infection of the Mouth.** At least thirty species of micro-organisms flourish in the oral cavity, some of them being pathogenic, staphylococci, streptococci, pneumococci, and often diptheria bacilli. They are constantly being washed away with the saliva, and through the extensive desquamation of the epidermis occasioned by mastication. Saliva is not always germicidal, but has an inhibitory reaction and weakens the virulence of some bacteria. The fetid breath and sordidity, observed in fever patients where the mouth is dry, may be attributable to the lack of saliva secreted and its consequent sterilizing qualities. The great rapidity with which wounds of the mouth heal is an important factor in the absence of serious infection from the wounds often occurring in this cavity.

**Protection of Wounds with Zinc Chlorid.** Brose's researches were conducted on rabbits and demonstrated convincingly that a wound cauterized with a fifty-per cent solution of zinc chlorid is protected against infection. Even with extremely-virulent infection, and when an interval of a minute had elapsed between the wound and the cauterizing, the animals were absolutely protected. Notwithstanding this his researches failed to show that zinc chlorid can be regarded as a disinfectant, even in the strongest solutions. His researches further demonstrated that the results of infection differ accordingly as the germs come in light contact with the wound or are driven forcibly into it.

**Camphor as an Anesthetic.** Leredde, in *Journal de Medical Interne,* recommends the following combinations containing camphor as local anesthetics in affections of the skin accompanied by itching:

R. Camphorae........................gr. xv
Olei amygdalae dulcis..............ℨiiss
M. Fiat unguentum. Sig.: To be applied locally.

Or R. Olei camphoratae....................ℨiiss

Chloralis hydratis....................gr. xv

Liq. petrolati..............................℥iii

M Sig. Apply locally.

Or: R Zinci Oxidi
Pulv. cretae
Olei camphoratae
Aquae calcis, aa..........................℥i
M. Sig.: Apply locally to the affected areas

**Hair Ball in Stomach.** Ranzi recently had occasion to remove a concretion of hair, threads and rubber bands from the stomach of a young woman. The concretion nearly filled the stomach and duodenum down to the ascending portion, and when removed presented a perfect cast of these organs, weighing after drying, 440 gm., and measuring

22 cm. in the stomach and 44 cm. in the duodenum, or 66 cm. in all, about 24 inches. He has found in the literature fifteen similar cases, eleven of which were operated on, with the recovery of all but one of the patients. The symptoms in the present case were referable to the stomach, and there was a history of the passage by rectum of a hair ball four years before, and a habit of swallowing hairs, threads, etc., since childhood. The movability of the tumor also aided in the differentiation. One surgeon removed a hair ball through a lumbar incision.—*Jour A. M. A.*

**The Books of the Year 1904.** Those who were interested in the summary of the new books published during the year 1904 which we gave in the *Journal A. M. A.*, January 7th, will be further interested in the following from the *Medical Book News*. Its summary includes new editions and re-publications. "Were any reader of this magazine, possessing $1,489.58, desirous of expending it in the purchase of new books for his library, every standard American and English work on medicine in each of its various branches, and on dentistry, pharmacy, chemistry and the allied sciences, published during the current year, could be included. This would mean the acquisition of 575 titles, containing 193,094 pages, with 40,689 illustrations, many in colors. Reducing the different values to a like basis, the cost of each octavo book of 335 pages and 70 illustrations would cost an even $2.59."

**Total Abstainers and Life Insurance.** Jews are not the only long-lived class, according to the New York actuaries. While the well-to-do Hebrew who takes life insurance is said to be 15 percent better risk than the average total abstainers from alcoholic drinks make still better showing—from 20 to 50 percent over the moderate drinkers, including the Jews. The average Jew is probably not a total abstainer, and it would be of interest, if it were possible, to study out by comparison with the teetotalers the reason for the advantage of the latter. From 5 to 35 percent is therefore the percentage that can be credited in favor of abstinence. Of course, there are many elements that enter into the solution of the question beside the mere abstinence from alcohol, such as the probable relatively lesser frequency of accidents and exposure, etc. It seems pretty clear, however, that the advantage is with the total abstainers, and that even moderate drinking detracts from the tendency to longevity.—*Jour. A. M. A.*

**Ether versus Chloroform.** Conclusions deducted from a careful study of the literature of the last decade, in addition to experimental and clinical research, clearly indicate that chloroform depresses the heart, the respiration and the blood pressure, while ether, in the standard dosage, does not effect these functions. They add that nerve substance exposed to the fumes of chloroform is killed, while ether fumes induce only a transient paralysis. The safety zone is much wider with ether than with chloroform, and the former does not have such an injurious action as the latter on the parenchyma of the various organs when the narcosis is long protracted. The concensus of opinion seems to be that ether is consequently less dangerous than chloroform, and that the pulmonary complications sometimes observed consecutive to ether anesthesia are not so much the fault of the ether as of the anesthetist.—*Jour. A. M. A.*

**Pulp Mummification.** If the pulp is exposed, place a small quantity of nerve paste on the pulp and seal the cavity with cement for two weeks. If at that time the tooth is comfortable, open thoroughly to the pulp chamber and syringe thoroughly with warm sterilized water. Isolate the tooth with dam or cotton rolls and dry out the canals as thoroughly as may be desirable or practicable. Place in the pulp chamber a ball of mummification paste large enough to fill the chamber and canals and force to place with small burnishers, and fill the cavity with cement, gutta-percha or amalgam, as may seem best. The mummification paste is made of equal parts of acetanelid and powdered alum, to which enough glycerine has been added to make a paste of proper consistency for manipulation. A little oxid of zinc will make the paste more consistent, too much will however make it hard.—Dr. C. L. Marey, in *Brief*.

**Septic Cellulitis with Acetozone.** An old lady, aged 79 years, received a slight abrasion on the back of the right hand by knocking it against a screw on her kitchen table, as she was washing dishes. She said the hand began to itch immediately, and in half an hour to swell, and the pain was terrific and hard to bear. The family, not realizing the gravity of the condition, allowed the wound to go unattended for thirty-four hours before calling me.

Upon my arrival at her home the whole hand was enormously swollen, the tumefaction extending to the elbow. Recognizing the condition at once, I made three incisions in the hand and inserted into each of them a piece of gauze saturated with acetozone solution, and put on a large gauze and cotton dressing, directing the family to keep the whole saturated with solution at half strength. Forty-eight hours after the initial dressing the hand was nearly in condition for use again.—E. A. Crain, M.D.

**Igorrote Longs for Gold Teeth.** Believing that gold teeth were all that was needed to make him the envied society leader of the Igorrotes, Sudong, one of the ambitious members of the tribe, deliberately smashed two of his front teeth in order to have a pretext for going to the dentist.

Sudong's efforts were in vain, however, for Gov. T. K. Hunt, who is in charge of the Igorrote village, discovered the ruse and refused to allow the Igorrote to have his teeth crowned. Gov. Hunt believes that the cunning Sudong will find some other excuse and that ultimately he will obtain the coveted gold filling.

To the instinct of imitation, which is strong in semi-civilized people, is to be attributed Sudong's desire for gold-filled teeth. According to the statement of Gov. Hunt, Sudong first got his idea of having his teeth crowned with gold from the many crowns he had seen in the teeth of visitors to the Igorrote village. He believed that Americans filled their teeth with gold as an ornament, and he determined to do likewise.—*American Journal.*

**Cut Rate Drugs.** New York police believe that they have discovered a gigantic drug swindle, and several arrests have been made on information that well-known proprietary medicines were being counterfeited and sold as genuine. It is stated that slips were found in the places raided, giving the names of 5,000 druggists throughout the country to whom dangerous mixtures, made in New York, were sold. It is further stated that circulars sent out to druggists and containing the code by which they could order the preparations were found. The price list showed that the drugs in the list were being sold at from 15 to 50 percent below the usual selling price. It is inconceivable that the regularly licensed retail druggists, who usually buy from responsible wholesalers, were victimized to any great extent by this swindle. Perhaps this explains, however, where department store and other price-cutters have been getting their goods and also the inability, in many cases, of responsible manufacturers to deal with the price-cutting evil.—*Pacific Drug Review.*

**Investment Material.** About five years ago I was convinced that crowns and bridges could not be properly constructed without using one of the various investment compounds that we see advertised, but I have re-adopted the old-style-sand-and-plaster investment with very pleasing results. Some time ago I procured a peck of sand such as the cement-walk builders use, and when I have occasion to invest a case I employ the following method: First add a little potassium sulfate to the water in the plaster bowl and sift in the amount of plaster necessary for the case. After the plaster is absorbed pour off the surplus water, and stir in sand until the mixture looks like the mortar that is used for plastering houses. Place this upon a glass slab and invest the case. It is better to trim off the surplus while the investment is soft, for when it becomes hard it is difficult to do so. This investment will be ready for the fire in three minutes or less, and I do not know of any material that will stand as much heat and be as hard after heating. It is simple and cheap, and I hope that others may take the pleasure in its use that I have had.—E. H. Allen, in *Dental Review.*

**Mechanical Purification of Milk.** Diffloth reports that great progress has been realized by the cotton filters, a Swedish invention, now used for purifying milk. The milk is strained through two wire strainers holding between them a flat, round, small sheet of cotton. The cotton is thrown away and replaced by a fresh disc of cotton after using. In eight months of tests with this cotton filter his inoculation of animals with the milk never caused morbidity, and the milk thus filtered could be kept for many days without souring. Diffloth regards the results of these tests as very remarkable in regard to the preservation of the chemical composition and of the digestibility of the milk, but as the milk was obtained from a model dairy they are not conclusive in respect to the microbes. Centrifugation has no effect on pathogenic germs in milk, or is liable to favor their proliferation. He adds that stock raisers have found it necessary to pasteurize "skim milk" from the creameries before feeding it to their pigs, as it is much harder to preserve than "skim milk" from natural cream rising. Centrifugation also interferes with what he calls the stereochemistry of the milk, that is the molecular arrangement of its constituents, and it has the further disadvantage that it expels the gases.—*Press Medicale.*

**Oxygen in Surgical Infections.** Dr. Thiriar recently communicated to the Belgian Academie de Medecine his further successful experience with the direct application of a stream of oxygen to infected tissues. He uses it in the form of a permanent application—a tube is inserted in the lesion, communicating with oxygen tank, and left undisturbed for several days. Every case of infection as a serous membrane has been benefited by this treatment in his experience, but the results have been most striking in gaseous septicemia. The oxygen not only stimulates the tissues and promotes phagocytosis, but also kills the germs, substituting an oxygenated emphysema for the microbian emphysema. Oxygen applied under pressure to a furuncle or carbuncle has always aborted or cured it in a few days, and it has proved its usefulness in hundreds of cases of diffuse phlegmons, gangrenous erysipelas, suppurating complicated fractures and arthritis. He does not advocate it for generalized infections, although recent publications have proclaimed the feasibility of intravenous injections of oxygen, with which Thiriar has had no experience.—*Jour. A. M. A.*

**Amalgam Crown.** A badly decayed lower first molar was excavated and roots filled, the crown being entirely gone. A staple was then made of heavy platinum wire, one prong projecting as far as practicable into each root, the bow standing up two-thirds of the occlusal space. A band of thin German silver was then made to almost actual occlusion with the upper teeth and contoured to the adjacent teeth and fitted to place. A piece of soft wax was then forced into the band so as to get an impression of the end of the roots and left projecting to occlusal edge of the band. A modeling compound mashbite was then taken of the adjacent teeth of both jaws, and this was run with plaster. When set, the modeling compound was removed, also the wax in the band, leaving the band with the staple fastened into the plaster. The cavity in the band was then filled with amalgam and contoured to occlude with the upper teeth. When the amalgam had set, the German silver band was removed and the amalgam crown polished and set with cement. It made a good, strong crown and was perfectly adapted.—J. H. Brown, in Sept. *Brief*.

Time could have been saved by packing the amalgam in the mouth and contouring to occlude with the upper teeth and then remove when set for polishing.—ED. REGISTER.

**A Remarkable Fitting Plate.** One of the most peculiar of my experiences was in the mouth of a woman who came from a country town. She was wearing a partial denture of gold clasped to the natural teeth. A clasp on one side had become broken and she had come down to the city to have the plate repaired.

The plate was very foul, and when removed from the mouth was carried at arm's length and placed in a dish of sulphuric-acid pickle for boiling. The plate was repaired by the addition of a new clasp and was inserted in the patient's mouth.

"You would better remove it and again place it in position," said I, "to see that it is all right and that you will be able to manage it by yourself, because you must take it out and clean it after each meal."

I used this method to instruct her as to proper cleanliness.

Her answer in her brogue was as follows:

"Shure, I'll not take it out at all. Whin I had the plate first made I wint up to me home in Calistogy and like a fool I tuk thim out and I couldn't put thim back agin, so I had to come clear down to the city agin to have the dintist put thim back. That was sivinteen years ago and I niver had thim out of me mouth since."—Dr. Knowles in *American Journal*.

**Glass Points for Burnishing Matrices.** Quite a number of years ago I had occasion to call the attention of this society to certain glass points for the packing of gold. I have used those glass points from that time to this, only using them against very thin walls, where pressure would not be permissible, and they work very well. They do not catch on the gold as the ordinary steel burnishers do. They were suggested to me by the use of Dr. Shumway's ivory points. Since the method of making our inlays came into use, I have used those glass points for burnishing the gold and the platinum, and they are very nice for that purpose. In the presence of several gentlemen, a few nights since, I had occasion to speak of them, and to my surprise no one present seemed to know of them. So I thought it might be worth while to call attention to them again, because they can be so easily made by any one, and they are very valuable for that sort of work. They are made of pieces of glass tube, which you can get at the druggist; put one end in a blow-pipe flame, soften, and draw out; then hold in the flame and a little ball rolls up on the point. This makes a nice ball burnisher. You can get a variety of shapes to the points, so that they can be adapted to various positions in the mouth. Of course, the material we use—the thin gold or thin platinum—is so very soft and plastic, and requires so little force, that they do not break when put to such use. They are so very smooth that they do not catch and drag on the gold, as the steel burnishers do.—Dr. S. G. Perry, in *Cosmos*.

**Damage Suits.** The Superior Court of California decided last month against a dentist. He sued the proprietor of a drug store for $5,000 damages, alleging that he ordered a bottle of still water, but was given a bottle of highly-charged water, and that the bottle broke and cut one of his hands, impairing the use of it. The court decided that the defendant was not responsible for the accident The Supreme Court of California last month affirmed the judgment of the Appellate Court in assessing $2,000 damages against a dentist, for injuries resulting from the alleged unskillful extraction of a woman's tooth. A woman in St. Louis has sued a dental college for $10,000 damages, alleging that while having a tooth filled at the college one of the operators drilled through the root, injuring her gum so that she was unable to attend to her duties. This month a man at Croton, O., sued a dentist for $200 damages, alleging that the dentist dislocated his jaw while extracting a tooth. The jury found no cause for action and the case was dismissed. This month a woman at Bayonne, N. J., swore out a warrant for a dentist, alleging assault. She claimed that because she did not have sufficient money with her to pay the small balance due on her account the dentist refused to allow her to go or to send for the money and removed a bridge which he had just set in her mouth. This month a dentist in Philadelphia was sued for $1,000 damages by a man who claimed that the dentist extracted a tooth in such an unskillful manner as to seriously injure the jaw. A Memphis, Tenn., dentist was find $25 and court costs for removing a gold crown from a woman's mouth because she did not have enough money with her to pay the balance due on it.—January *Digest.*

**Mistaken Diagnosis.** A healthy man of about thirty-five had a discharge upon the jaw opposite the bicuspid or molar tooth for a year or more. He had consulted his family physician, who told him that his blood was diseased, and had given him remedies. He was obliged to wear a black patch for the discharging sinus. He was traveling one day in one of the New England States, and a gentleman sitting behind him leaned forward and said, "My friend, that comes from a tooth." He said, "You are mistaken; my physician in Philadelphia tells me it is from the condition of my blood." The reply was, "Well, I am a surgeon. I simply say that comes from a tooth." The gentleman was a little indignant, but when he came to Philadelphia he consulted Dr. Agnew, who said, "I think he told you the truth," and referred him to me. The gentleman brought his dentist with him for a consultation. The dentist began by saying, "I know, doctor, this does not come from his teeth; I have examined them over and over and over again." I looked into the patient's mouth and opened the right lower second bicuspid. I passed in a probe, and pus welled up. The dentist had been for thirty years in practice and had never seen one of these cases. I advised extraction, which was done, and three days later I met the gentleman, who said there had not been a particle of pus since.

I mention this to show that a dentist with thirty years of experience may never happen to see an abscess discharging at the angle of the jaw from a lower molar or bicuspid tooth, and when he has it before him he may not recognize it. One can hardly conceive of that happening. The surgeon in this instance was brighter than the dentist.—Dr. Darby, in *International.*

